# Hierarchical growth in neural networks structure: Organizing inputs by Order of Hierarchical Complexity

**DOI:** 10.1371/journal.pone.0290743

**Published:** 2023-08-31

**Authors:** Sofia Leite, Bruno Mota, António Ramos Silva, Michael Lamport Commons, Patrice Marie Miller, Pedro Pereira Rodrigues

**Affiliations:** 1 CINTESIS – Center for Health Technology and Services Research, Porto, Portugal; 2 Dare Association, Inc. Boston, Massachusetts, United States of America; 3 Laboratory of Experimental Mathematics and Theoretical Biology, Physics Institute, Universidade Federal do Rio de Janeiro, Rio de Janeiro, Brasil; 4 Department of Mechanical Engineering, Faculty of Engineering University of Porto, Porto, Portugal; 5 INEGI Institute of Science and Innovation in Mechanical and Industrial Engineering, Porto, Portugal; 6 Beth Israel Deaconess Medical Center, Harvard Medical School, Cambridge, Massachusetts, United States of America; 7 Department of Psychology, Salem State University, Salem, Massachusetts, United States of America; TU Wien: Technische Universitat Wien, AUSTRIA

## Abstract

Several studies demonstrate that the structure of the brain increases in hierarchical complexity throughout development. We tested if the structure of artificial neural networks also increases in hierarchical complexity while learning a developing task, called the balance beam problem. Previous simulations of this developmental task do not reflect a necessary premise underlying development: a more complex structure can be built out of less complex ones, while ensuring that the more complex structure does not replace the less complex one. In order to address this necessity, we segregated the input set by subsets of increasing Orders of Hierarchical Complexity. This is a complexity measure that has been extensively shown to underlie the complexity behavior and hypothesized to underlie the complexity of the neural structure of the brain. After segregating the input set, minimal neural network models were trained separately for each input subset, and adjacent complexity models were analyzed sequentially to observe whether there was a structural progression. Results show that three different network structural progressions were found, performing with similar accuracy, pointing towards self-organization. Also, more complex structures could be built out of less complex ones without substituting them, successfully addressing catastrophic forgetting and leveraging performance of previous models in the literature. Furthermore, the model structures trained on the two highest complexity subsets performed better than simulations of the balance beam present in the literature. As a major contribution, this work was successful in addressing hierarchical complexity structural growth in neural networks, and is the first that segregates inputs by Order of Hierarchical Complexity. Since this measure can be applied to all domains of data, the present method can be applied to future simulations, systematizing the simulation of developmental and evolutionary structural growth in neural networks.

## Introduction

Scientific evidence shows that there is hierarchical self-organization in the neural structure of the brain, namely, in how it is anatomically organized [1, 2], in its developmental dynamics throughout cognitive stages [3–6], in its developmental dynamics throughout neuroanatomic layers of neural complexity [7–13], from the perspective of complex systems [14–18], and from an evolutionary perspective [19–22]. This hierarchical dynamics greatly improves the adaptation potential at a low expense [23, 24]. The motivation behind the present work is to explore whether a neural network model can also exhibit hierarchy in its structural growth. In order to do that, neural networks will be presented with segregated subtasks of increasing order of hierarchical complexity (OHC), so as to simulate a developmental environment. By simulating a developmental environment, we are compelling the networks structure to respond developmentally as well, by increasing their complexity without replacing previous structures. This method here employed is an architectural method, a class of methods usually applied for addressing the problem of catastrophic forgetting in neural networks, which completes the motivation of this study. Hierarchical complexity is defined according to the Model of Hierarchical Complexity (MHC), and is a measure of complexity that can be applied to different domains of data. This is advantageous for exploring the simulation of developmental and evolutionary structural growth in neural networks in different domains of data and extract transversal and transdisciplinary conclusions. The present work demonstrated that neural networks exhibit progression in its structural complexity growth across developmental problem solving, when subtasks are defined by the OHC. The challenge of this work was to search of the minimal complexity connectionist structure that solves each increasingly complex problem, such that a structural progression approximates a fair modelling of the developmental phenomena [25].

### The Order of Hierarchical Complexity

The Model of Hierarchical Complexity (MHC) is a post-Piagetian mathematical model of human development and evolution. It defines the Order of Hierarchical Complexity (OHC) as an a-priori measure of difficulty of tasks. In the present work, increasingly complex tasks were operationalized using the measure of OHC. The OHC corresponds to the number of recursions necessary to reach the basic elements that compose the task. It can be used to assess the stage of development of individuals, which can be humans, non-human animals or computational agents. Hence, an OHC can be in principle attributed to all tasks in all domains of knowledge [3]. The relationship between the OHC and stage of development is a one-to-one correspondence: the highest OHC task an individual can successfully solve, independently of the domain, dictates their stage of development. 17 OHC and 17 stages have been found experimentally, by observing the behaviour of individual, with equal gaps between orders [26]. There is evidence that the OHC of a problem predicts performance (stage), which correlates with Rasch coefficients (*r*’s). Here, *r* is the result from Rasch analysis in a scale ranging from 0 to 1, which has been found to lie between 0.90 and 0.98 on a variety of different tasks and domains [27].

In terms of development, the MHC shows that individuals proceed along the sequence of 17 stages, always starting out at stage 0, but not necessarily achieving stage 17. Actually, the highest stage attained varies across individuals. During infancy, healthy individuals proceed fast approximately along the first 8 stages, and then slow down the developmental pace. The best curve fitting found to describe this developmental trajectory is a logarithmic curve that varies with age [28]. Following this discovery, the MHC provides evidence that stage of development is imprinted in the neural structure since birth and hypothesizes that each stage has a neural signature [11, 28]. This means that perceiving a task of a given OHC triggers the activation of a given neural structure. This indicates that organizing the world by OHC is a trigger for uncovering the hierarchical nature of organization in living organisms, which justifies our methodological approach.

In terms of stage transition, it is widely accepted that a higher-order stage is more complex than the immediately previous lower-order stage, and that a higher-order stage emerges out of the previous in a self-organized way [26]. However, more precise dynamics of stage transition are still poorly understood. In the present work, we tested if segregating the input set by OHC would lead to observing a hierarchical growth of the structure of a neural network model. In order to not interfering with the structural organization of networks for adjacent OHC input sets, models were trained separately and independently for each OHC input. Only afterwards they were compared. Networks were trained to solve the balance beam problem, which is a developmental problem composed of different OHC, as will be detailed next.

### Related work

Currently, neural network models designed to solve complex problems are usually programmed with a complex structure built from scratch, which is usually employed for solving the complex problem from scratch [29–32] using enormous datasets [33], and tending to overfit [32]. They do not develop from simple to more complex algorithmic structures, or from simpler to more complex artificial reasoning abilities [34], as we observe in the human brain [1, 7, 9–11, 35, 36]. Generative architectures are an exception to fixed structures [37, 38], but newly generated structures overwrite the existing ones. In contrast, in the developmental trajectory of the natural brain, older simpler structures remain available for solving simpler problems, enabling an enormous adaptive flexibility [5].

In the past, experiments with developmental neural networks showed that it is important to think in developmental terms at every step of an algorithm, from inputs, to algorithmic structure, to outputs. This reasoning is also the basis of the present experiment: a neural network model that receives increasingly complex inputs and develops its structure to solve them. In regards to developmental inputs, pioneering experiments simulating natural language processing showed that a developmental network could only solve complex problems if it was first fed with a constrained input set [39], already simulating that more information is processed at each new stage. Nowadays, sparsity in training neural networks has been a choice for reducing the amount of computations per matrix [40], but this option is employed within the range of already complex models. In regards to a developing structure, experiments simulating date calculation were the first to show that increasingly complex portions of the problem should be solved by partially independent network structures hierarchically linked, roughly simulating stages of development [1, 9, 19]. Nowadays, this has been solved by augmenting the network structure [41]. For instance, a significant set of experiments replicating children’ behavior in the balance beam problem [42–44] tested several neural network architectures [37, 45–54].

In these simulations, generative architectures has been proven to be the best choice [34]. Here, new structural elements are recruited as they prove necessary for accommodating more complex portions of the problem. However, all these previous approaches are limited in the sense that when a more complex structure develops to solve an increasingly complex portion of the problem, previous and simpler structures are superseded and erased [34, 49]. This issue is related to the problem of catastrophic forgetting, for which lower levels or previous learning mappings get overwritten by newer ones, drastically impeding machine learning models to learn more than one task, to adapt to outliers, and to learn more complex portions of a task without forgetting simpler portions [55–58]. In contrast to catastrophic forgetting in often observed in machine learning, individuals can always resort to more elementary stages of development if it is sufficient, which comprises an enormous flexibility and adaptation potential [5]. This dichotomy leads naturally to the present work, inspired by the methodology of the MHC, and the first that segregates the input sets by OHC. Segregating by OHC is assumed to have two main advantages. First, since all tasks can be attributed an OHC, the segregation by OHC can be applied to all datasets. This provides for a systematization of knowledge through using neural networks as simulators of hierarchical growth. Second, segregating inputs by OHC potentially addresses the problem of hierarchical catastrophic forgetting in neural networks.

## Objectives

Our first objective is to explore the potential of neural network models to capture the notion of OHC, by exhibiting some form of hierarchical structural progression across orders. The second objective is to create a sequence of increasingly complex network model structures, where a more complex network is built out of the less complex, without replacing it. This work can be included in the category of architectural methods for approaching the problem of catastrophic forgetting in neural networks [55, 59], since we are trying to protect lower order structures from the interference due to the creation of higher order structures.

## Methodology

We simulated the Balance Beam test, a developmental test applied to children with different configurations of weights placed at different distances on each side of the fulcrum. Some configurations are more difficult than others, reason why some configurations are solved later in the developmental trajectory. The method employed in this work belongs to the class of continuous learning methods for neural networks, subclass of architectural methods. The method we propose requires three steps. First, we segregated the input set into disjoint subsets of sequential OHC. In other words, we segregated the balance beam test into subsets of increasing complexity, following the order in which they are solved in the developmental trajectory. Second, we trained independent neural network models for solving each sequential OHC and then compared models of adjacent OHC. The minimal complexity model for each OHC problem was searched. Third, we run sequential networks to solve the entire problem developmentally, by using a less complex network structure as the initial context for training the immediately increasingly complex structure.

### OHC input

**Fig 1:**
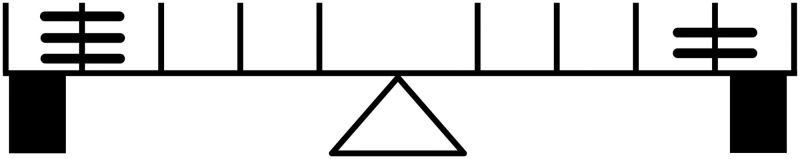
Concrete stage 9 balance beam configuration. Solved by using the counting operation.

**Fig 2:**
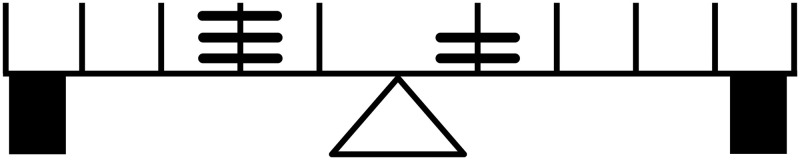
Abstract stage 10 balance beam configuration. Solved by using the sum operation.

**Fig 3:**
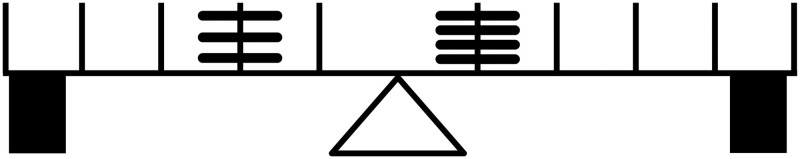
Formal stage 11 balance beam configuration. Solved by using the multiplication operation.

**Fig 4:**
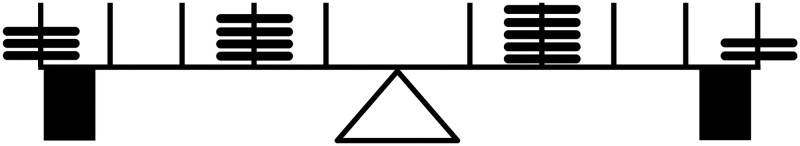
Systematic stage 12 balance beam configuration. Solved by using the distributive law operation.

The balance beam problem is a longstanding developmental test that has been created to assess 5 to 12 years old children’ cognitive development [60]. The most recent forms of the test consist of presenting the child a beam with a hinge in the middle, weights on both sides at various distances, and blocks underneath each extremity of the beam (represented in Figs 1–4). By changing the weights and their distance from the center, different test configurations are created with different levels of difficulty. When presented with a given configuration, children are required to predict the state of the scale if the supporting blocks were removed from below. The three possibilities of response are falling left, balancing, or falling right. Extensive research on developmental psychology has shown that children correctly respond to more difficult configurations of the test as they develop [43, 44, 61, 62].

There are different performance assessment schedules. The MHC is one of them. It clearly characterizes difficulty in the balance beam problem by the mathematical operations that should be used to correctly predict the result of a given configuration [43], such as counting, sum, multiplication, and the distributive law. The capacity to apply each of these operations is acquired at the following stages, respectively: concrete-stage-9, abstract-stage-10, formal-stage-11, and systematic-stage-12. In Concrete-stage-9 configurations, only weights or distances in each side change; the other dimension remains constant across sides. Hence, in these configurations, there are weight problems, only changing the weight at the same distance, or distance problems, only changing the distance at which the same weight s placed across sides. In Abstract-stage-10 configurations, both weight and distance change in both sides, but a correct prediction is made if distances and weights are summed in each side and then compared. In Formal-stage-11 configurations, again both weights and distances change in both sides, but a correct prediction can only be done if weight are multiplied by the distance at which they are placed on each side, and then compared. Finally, in Systematic-stage-12 configurations, there are two sets of weights at different distances from the centre in both sides, requiring that the distributive law is used for resulting in a correct prediction of the state of the scale. This arrangement of two sets of weights per side is suitable of concrete, abstract, and formal configurations as well, but an arrangement of one set of weights per side is not suitable for systematic problems. Examples of each type of configuration is show from Figs 1–4.

Input data representing all possible configurations of the balance beam problem were simulated in two input datasets: A and B. Dataset B was created to respond to the changes introduced by systematic-stage-12 problems (inclusion of two sets of weights and distances) (see Table 1), and to verify whether this change had an impact in network structures.

**Table 1:** Datasets A and B.

Dataset A	Dataset B
A′=weightrightweightleftdistancerightdistanceleft	B′=weight_1rightweight_1leftdistance_1rightdistance_1leftweight_2rightweight_2leftdistance_2rightdistance_2left

Representation of the elements that compose Datasets A and B.

Each dataset was then partitioned into subsets of increasing OHC. Dataset A contains the subsets of concrete, abstract and formal problems. All these problems are represented as 4 integer-element input vectors, where weight and distance values ranged from 1 to 20. The chosen range is only important for the generation of enough input data, given that what really matters for problem solving is the operation among values on each side and the structure necessary to correctly solve the problem. Dataset B contains the subsets of concrete, abstract, formal and systematic problems. All these problems are here represented as 8 integer-element input vectors, as shown in Table 1. Because the possible number of cases for the formal configurations would be very high, we decided to limit the range of weights and distances from 1 to 5.

Outputs were Boolean 3-element vectors that represented one of each three possible classes—fall right [0 0 1], fall left [1 0 0], and balance [0 1 0]. Each OHC problem for each set was partitioned into three datasets: training (70%), validation (15%), and test (15%). The number of cases per class differed per experiment. Results will include a reference to that.

### Creating network structures

Neural network models were trained to solve each OHC subset. Networks with feedforward connections were used first on dataset A and then on dataset B. The optimal number of layers and units per layer was tested. Afterwards, the best connectivity pattern for a given number of layers and units per layer was tested.

#### Network hyperparameters

Networks were created using the neural network toolbox available in MatLab^®^, R2016b. The following hyper-parameters are in-built functions of MatLab^®^, R2016b and were kept constant during the creation of network structures. As we are dealing with precise learned functions (and not generalization ability) we believe that the impact of overfitting is negligible; also, multiple runs are employed to assure learning is not stuck in local minima.

The sigmoid activation function was set to the hidden units and the normalized exponential function (softmax) was set to the output units. The Nguyen-Widrow initialization algorithm was used. Initial values differed each time the network is initialized. The gradient descent algorithm with adaptive learning rate (*lr*) was chosen, with initial *lr = 0*.*01*. During training, at each epoch *ep*, if $\frac{error\left(ep\right)}{error\left(ep-1\right)}\;>1,04$, then, the *lr* decreases (*lr*_*ep*+1_ = 0,7 × *lr*_*ep*_) and weights and biases of epoch *ep* are discarded. Otherwise, the *lr* increases (*lr*_*ep*+1_ = 1,05 × *lr*_*ep*_) and the weights and biases of epoch *ep* are kept [63]. The cross-entropy loss function (*CE*) was used, calculated as *CE* = −*t* × log (*y*), where *t* represents the expected output, or target, and y represents the output generated by the network given the current set of weights. A maximum of 10 validation steps or a minimum performance of 0.001 were set. As soon as one of these was reached, the network would stop the training process, whether or not it had converged to a solution.

#### Network dependent parameters

As mentioned, the number of layers and the number of units per layer was tested. The maximum number of internal layers was set to 2 and the maximum number of units per layer was set to 20. Afterwards, 5 different connectivity patterns were also tested. The testing procedure went as follows.

When using feedforward networks, units and layers were added iteratively and systematically. The networks started with a single neuron and undergone the training process. As the error stopped evolving, the accuracy of the network was assessed. If the accuracy was lower than 100% a neuron was added to the network, avoiding underfitting. If a network achieved an accuracy of 100%, no more neurons were tested, avoiding overfitting. This process occurred first on Dataset A and then on Dataset B. Neurons were added sequentially, one-by-one, to the internal layer, until the maximum of 20. If the problem hadn’t been solved by then, experiments with 2-layer networks started. In two-layer networks (also called hidden-layer networks), for each number of units in the first layer, units were sequentially added in the second layer, from 1 to 20.

Hence, units were added until one of two conditions was verified—the current network reached a learning accuracy of 100% or the number of units had achieved the maximum. In the case of one-layer networks, 20 different network structures were tested per OHC subset; in the case of hidden-layer networks, 400 different network structures were tested (20 units in the internal layer × 20 hidden units). Each network was repeated over 20 trials to compensate for the random generation of initial weights.

The maximum number of units per layer (N_hu_) was set to 20, based on the following heuristic equation $N_{hu}=\frac{N_{s}}{p\;\times \;\left(N_{i}+N_{o}\right)}$ [64], where *N*_*s*_ is the number of training samples, *N*_*i*_ is the number of input units, *N*_*o*_ is the number of output units, and *p* is an arbitrary scaling factor, usually set between 2 or 5 and 10, here set equal to 7. During the initial iterations, thus, empirically-based, it was determined that setting the scaling factor to 7 was appropriate, and that adding more than 20 units per layer, in both order problems, was not informative. The maximum number of units was, then, based on the above equation, but adapted to the current case. Important to mention is the fact that heuristic methods should be used only as a departure for subsequent trial and error search, as occurred in the present work.

**Fig 5:**
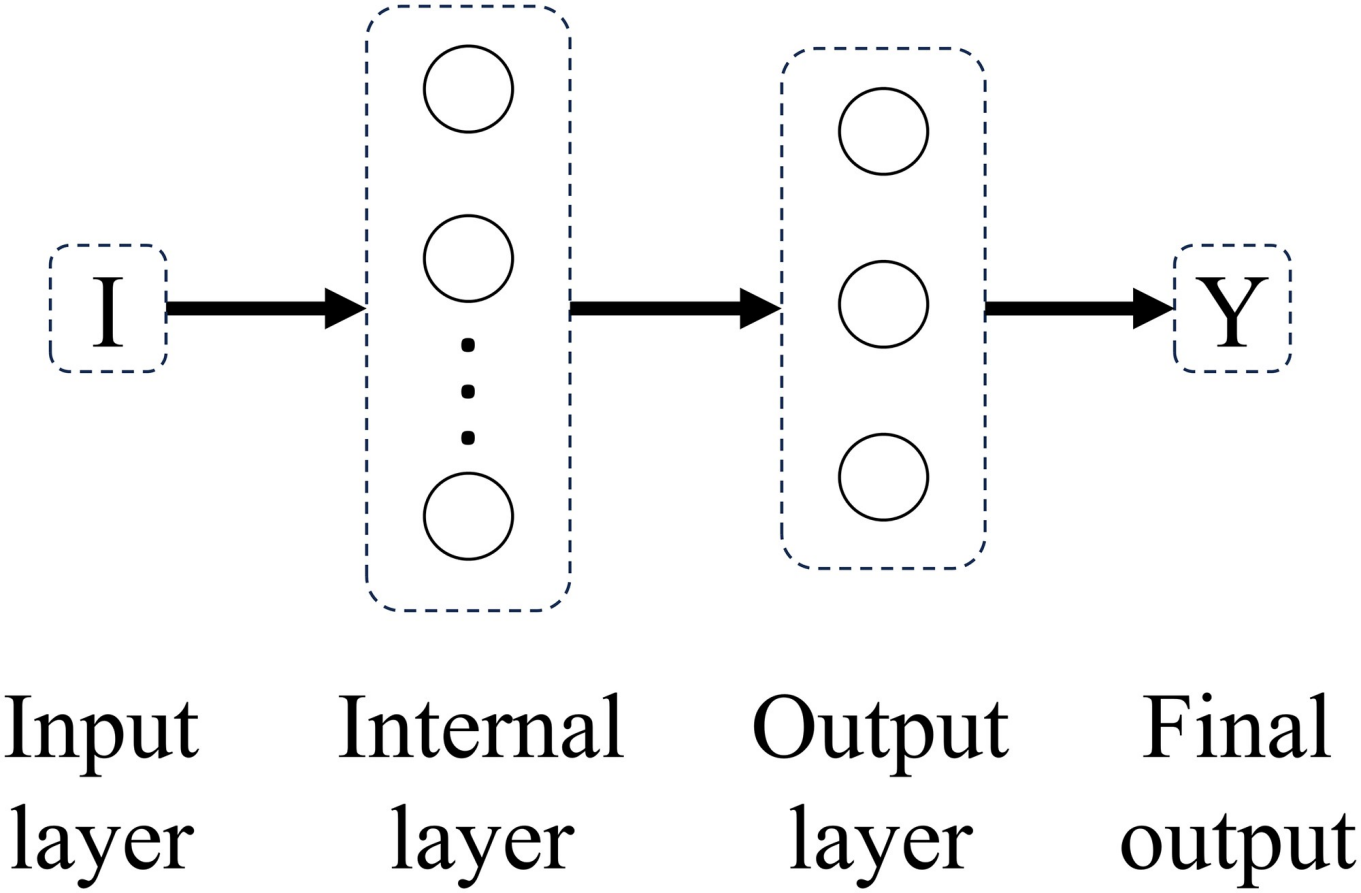
Network 1. One-Layer Network with Feedforward Connections.

**Fig 6:**
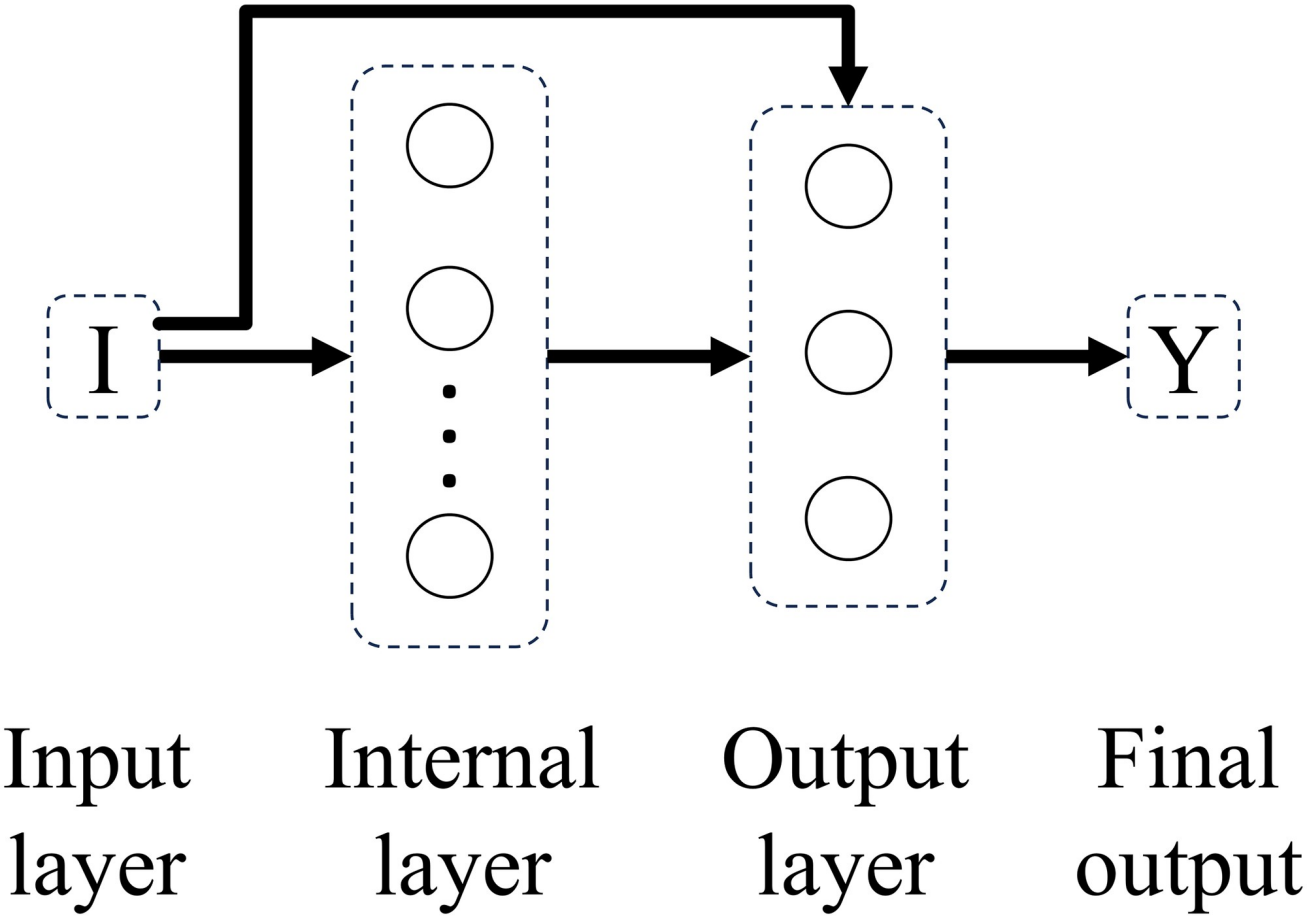
Network 2. One-Layer network with Input Connectivity.

**Fig 7:**
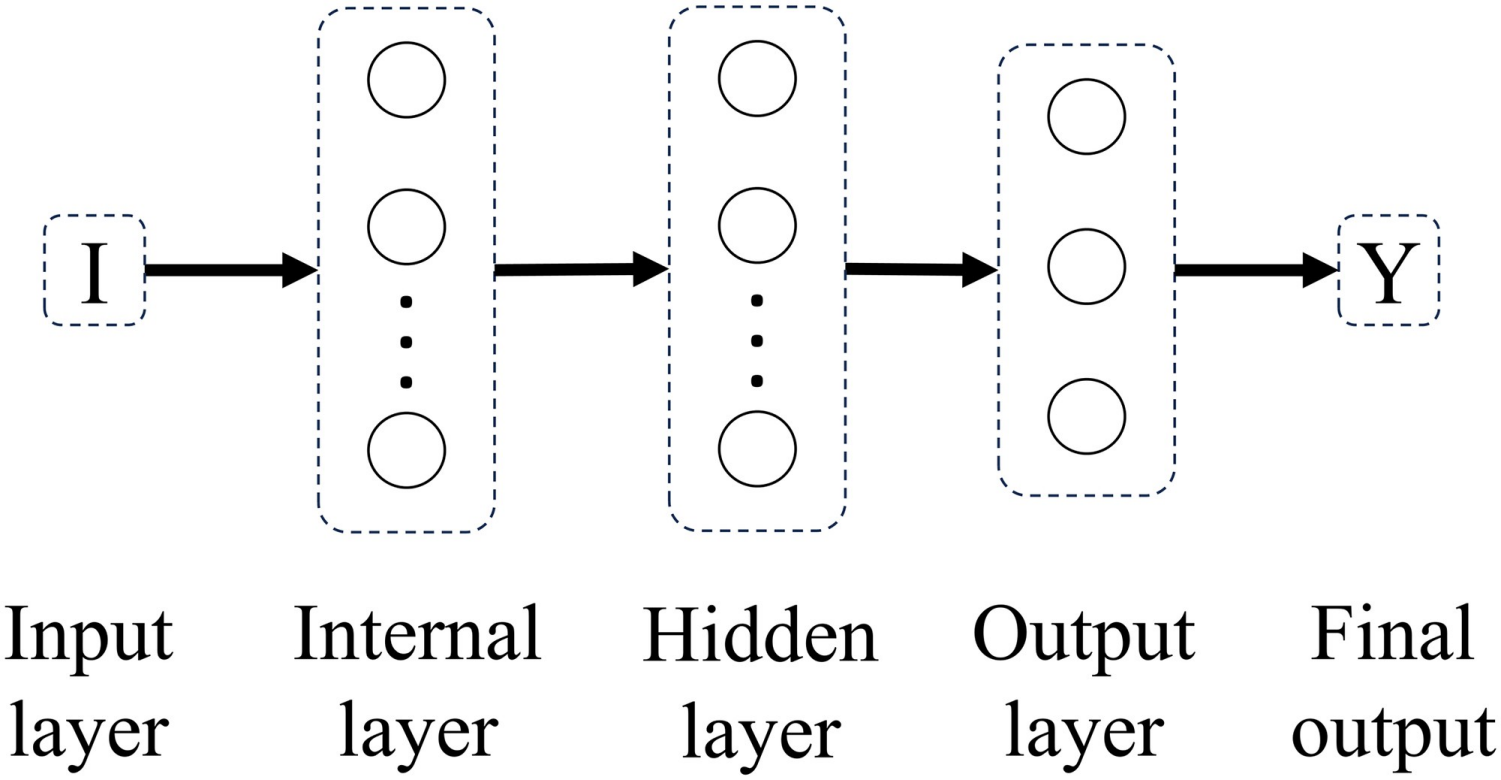
Network 3. Hidden Layer Network with Feedforward Connections.

**Fig 8:**
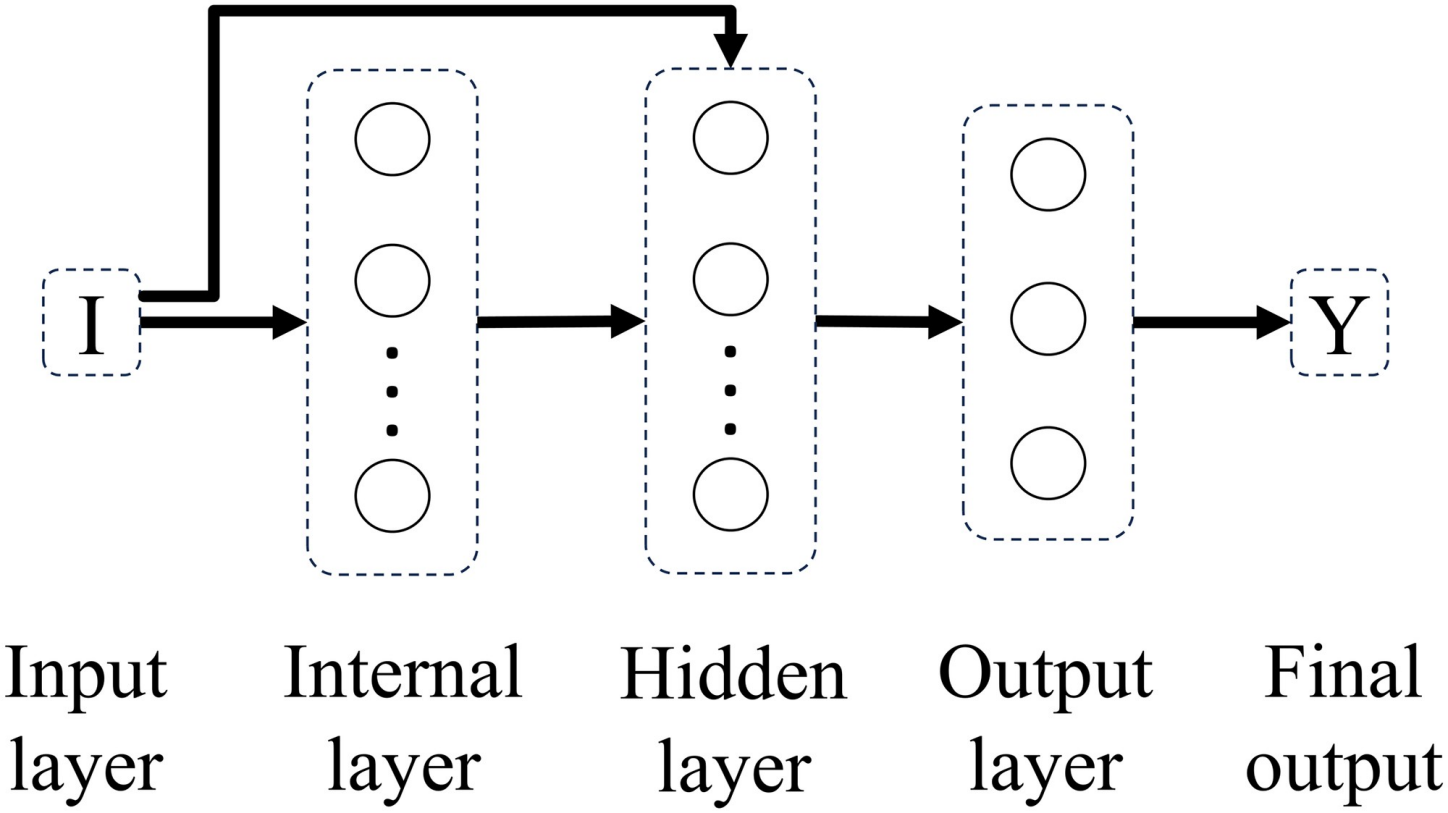
Network 4. Hidden Layer Network with Not-fully Input Connectivity.

**Fig 9:**
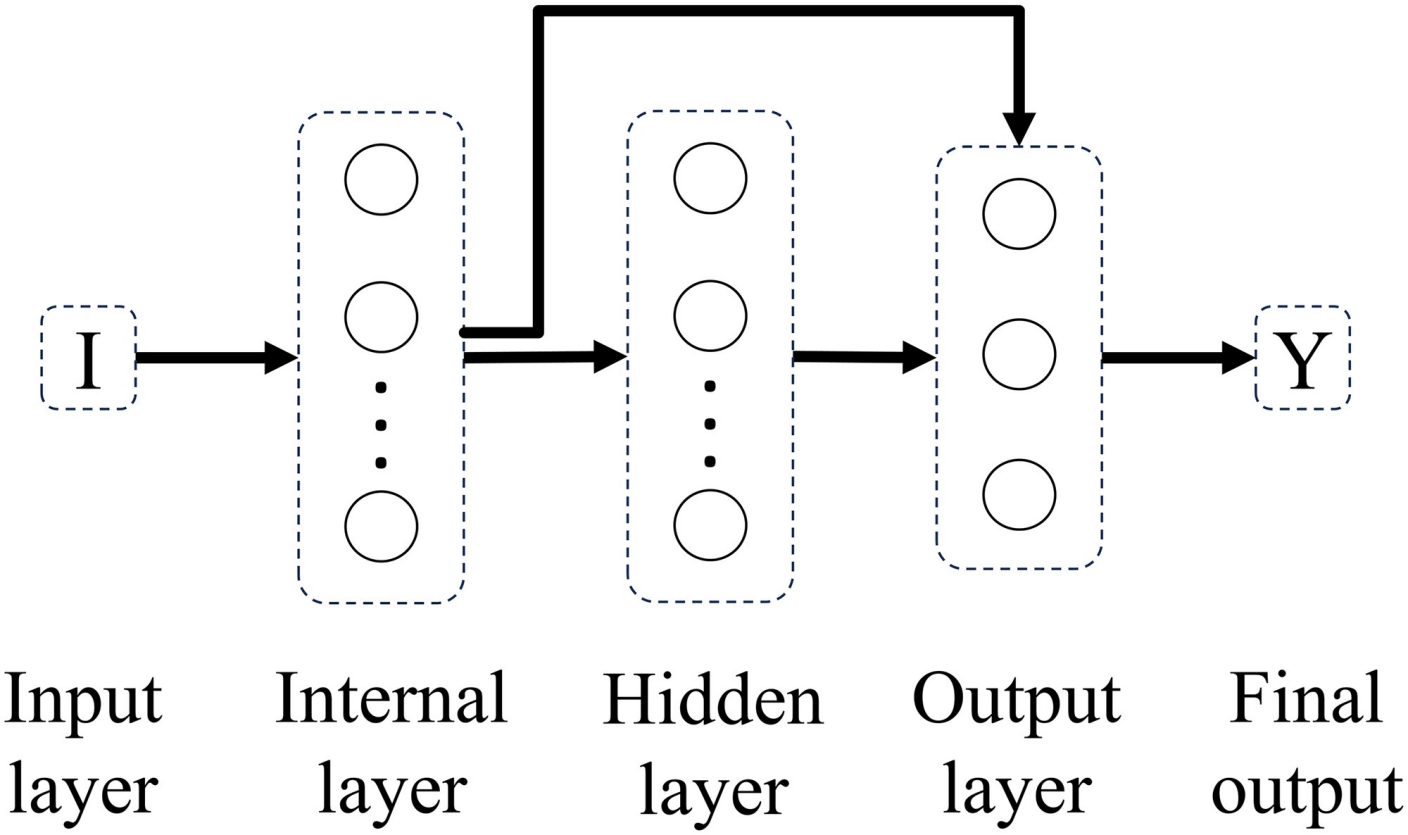
Network 5. Hidden Layer Network with Internal Connectivity.

**Fig 10:**
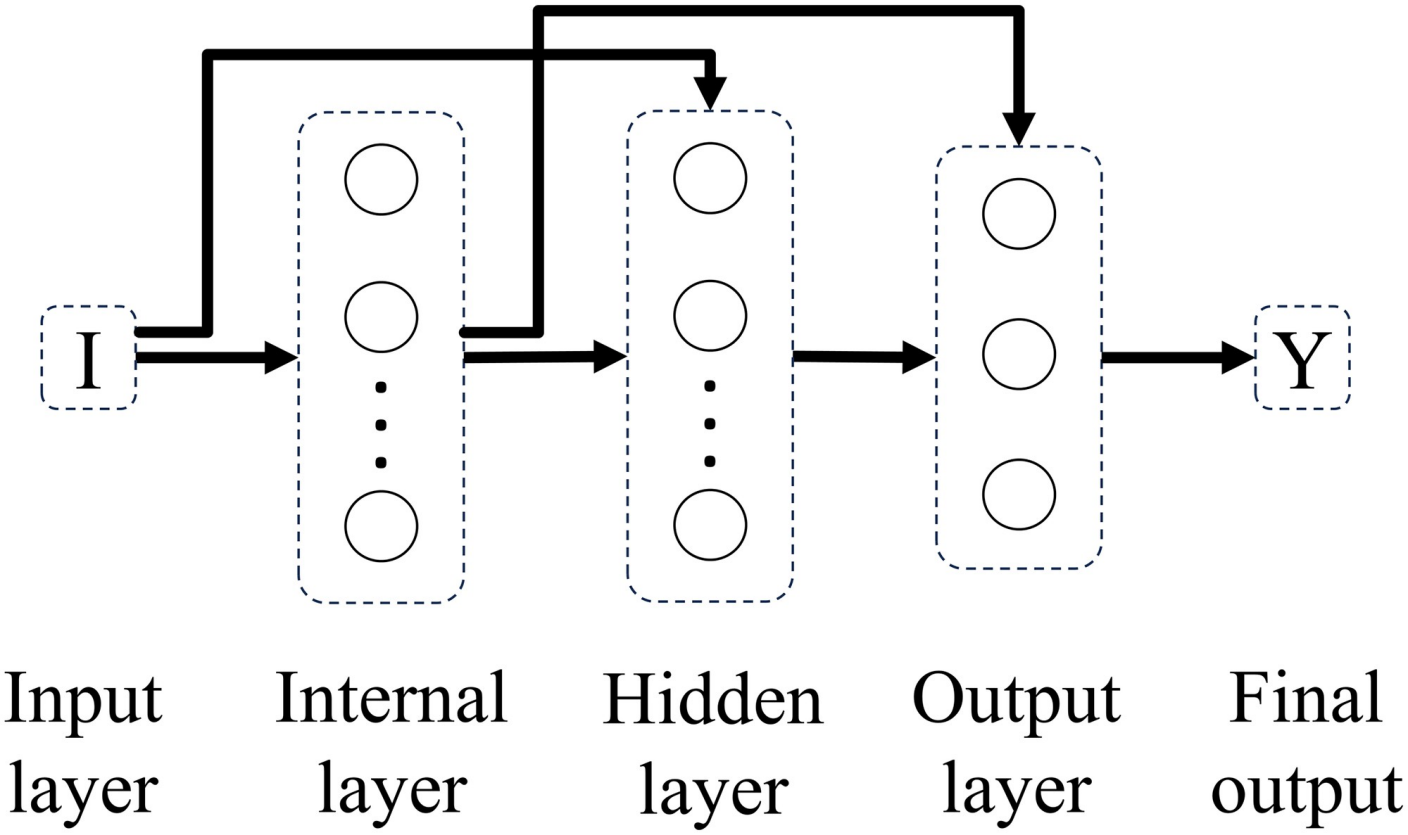
Network 6. Hiden Layer Network with Not-fully Input and Internal Connectivity.

**Fig 11:**
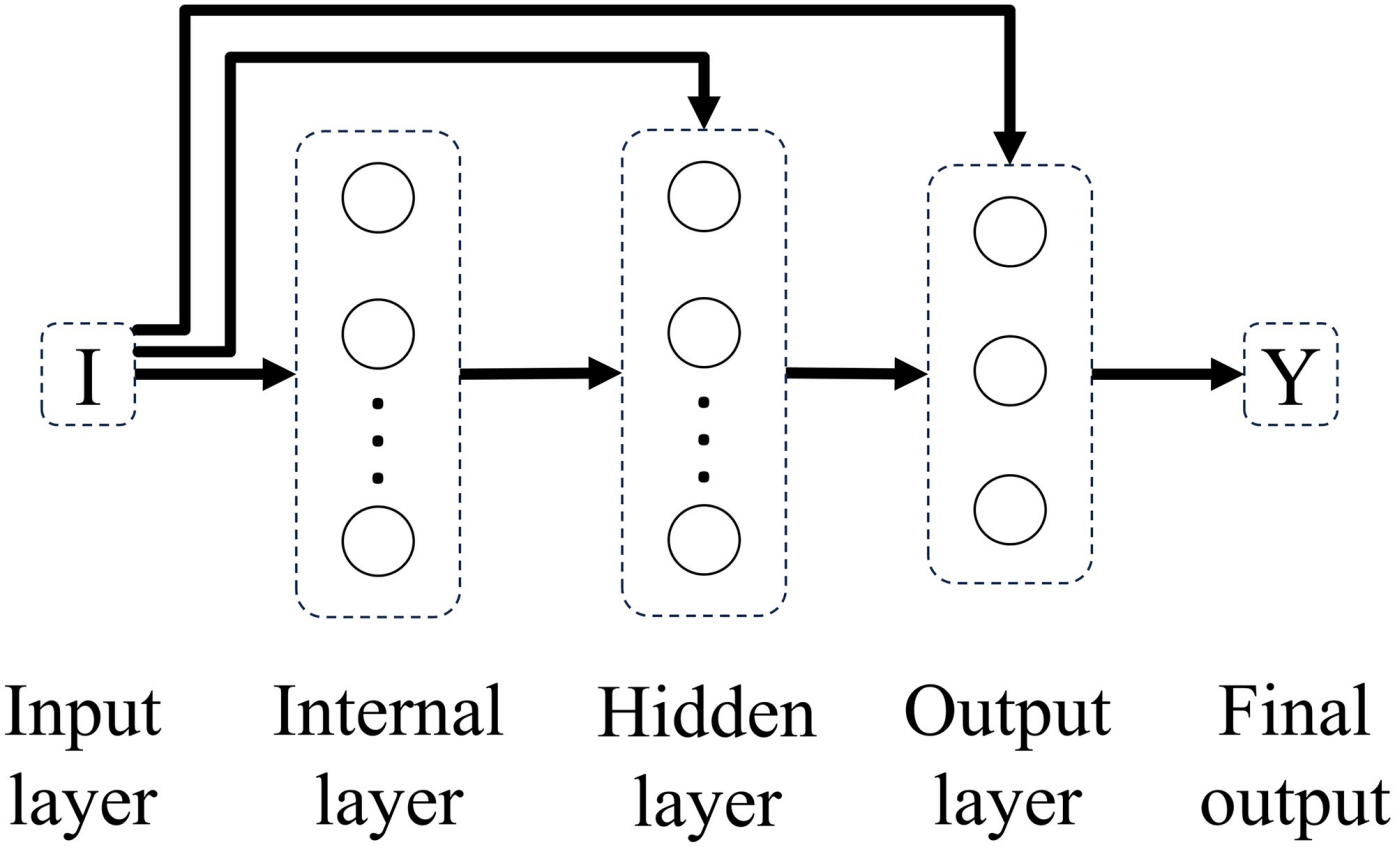
Network 7. Hidden Layer Network with Fully Input Connectivity.

Experimenting with different connectivity patterns followed. Whereas in a feedforward network all units in a layer are connected to all units in the subsequent layer, here, on different connectivity patterns, all units in a given layer are connected to all units in another given layer besides the subsequent layer. The only exception is backwards connections. Connectivity patterns are represented from Figs 5–11. For simplicity, arrows in the figure only display connections among layers, representing connections among the units that compose those layers.

Based on the results with feedforward networks, connectivity patterns were only tested for the subsets of formal-stage-11 and systematic-stage-12 problems (Dataset B). One-layer and hidden-layer networks with all 5 possible connectivity patterns were trained. Potentially, 20 perceptron networks (1 to 20 units in the internal layer) per connectivity pattern (5 types), and 400 hidden-layer networks (20 units in the internal layer × 20 units in the hidden layer) per connectivity pattern (5 types) would be tested, resulting in a total of 2100 different network structures. For systematic-stage-12 sub-problems, only hidden layer networks were trained, resulting in a total of 2000 different network structures.

#### Network performance

The performance of all trained networks was evaluated based on the following metrics. Mean accuracy (*A*_*m*_) is the mean accuracy of a given network structure, averaged across each set of 20 trials. Accuracy is measured as the ratio between the number of correct predictions and the total number of input samples. Maximum accuracy (*Max*_*Ac*_) refers to the maximum accuracy reached by a network of a given structure. Mean error (*e*_*m*_) refers to the number of wrong predictions outputted by a network of a given structure, averaged across each set of 20 trials. Total number of connections (*N*_*c*_) concerns the entire network structure, including input and output connections. Inverse measure of efficiency (*EF*_*hu*_) identifies the optimized point between increase in accuracy and increase in computational cost as units were added, and was calculated as ${EF}_{hu}=\frac{1}{N_{c}\times \;{e_{m}}^{1.5}}$. This measure represents the inverse of the network efficiency per number of units, where *hu* is a network identifier (*h* = number of internal layer; *u* = number of units in the last internal layer), and *e*^1.5^ represents the mean error of the network, potentiated to an arbitrarily defined parameter of 1.5. This parameter overweights an increase in accuracy against an increase in number of units and connections, such that successes are reinforced. Difference in *EF*_*hu*_ (Diff_EF_) refers to the variation in *EF*_*hu*_ as units are added, and was calculated as *Diff*_*EF*_ = *EF*_*hu*_ − *EF*_*hu*−1_. Negative values of Diff_EF_ correspond to an increase in *EF*_*hu*_.

After networks have been trained for a given OHC problem subset, accuracy prevailed over efficiency for the selection of the best network structure. If one network achieved an accuracy of 100%, it was selected. Otherwise, the selection of the best network structure, or structures, was done by inspecting the values of *Diff*_*EF*_ and choosing the most negatives ones. In this case, several structures could be candidates for selection. The network structures mentioned in the results are the candidates. Nonetheless, some aspects of this selection method will be object of discussion further ahead.

### Protecting lower-order structures

A final experiment was conducted to evaluate whether previous structures would get replaced by higher-order structures or not. The final weights of the network trained for a lower OHC problem were set as initial weights of the network for the next OHC sub-problem. At each transition, i.e., when a higher-order structure started to be trained based on a lower-order structure, four conditions of learning rates (LR) were tested. In three conditions, the learning rate applied over lower-order weights decreased by 20%, 30%, and 50%. In the fourth condition, these weights were not allowed update.

## Results

**Fig 12:**
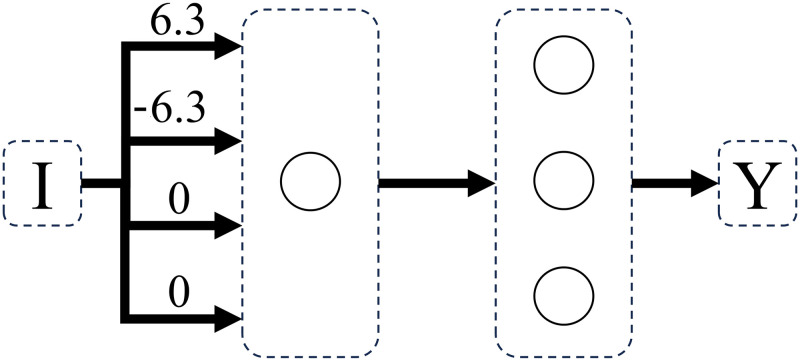
Network solving concrete stage 9 weight problems. Weight problems using counting operation (where only weight changes on both sides), using dataset A.

**Fig 13:**
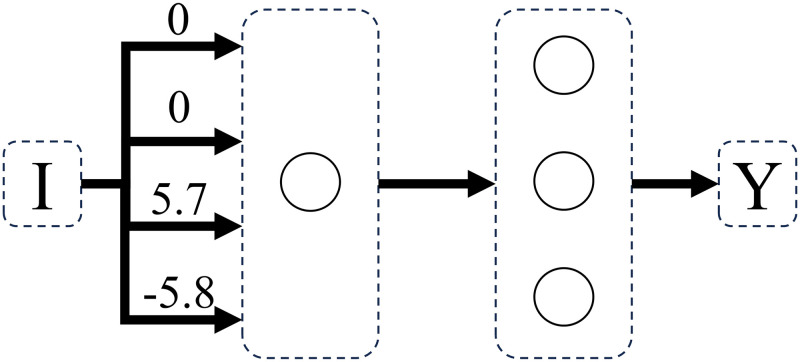
Network solving concrete stage 9 distance problems. Distance problems using counting operation (where only distance changes on both sides), using dataset A.

**Fig 14:**
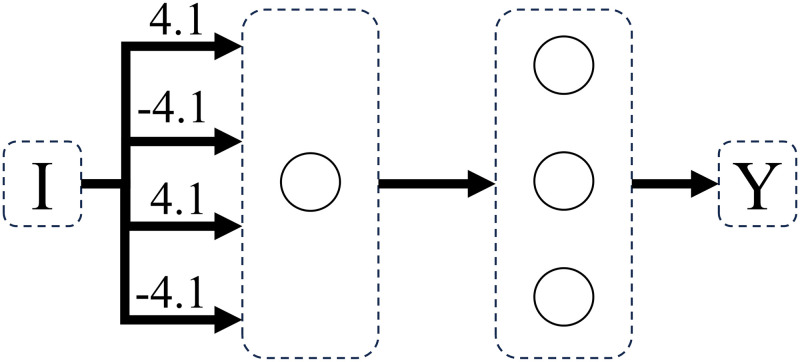
Network solving abstract stage 10 problems. Sum problems (where weight and distance changes on both sides), using dataset A.

Concrete-stage-9 and abstract-stage-10 problems were learned with 100% accuracy by perceptron networks with feedforward connections and one computational unit. In both Dataset A and Dataset B, the transition from concrete-stage-9 to abstract-stage-10 was made by a superimposition of weight matrices (Figs 12–14), where only the number of active connections increased, not the quantity of nodes in the structure. This represents the fact that, at abstract stage, the value of both weight and distance dimensions changes.

The transition from abstract-stage-10 to formal-stage-11 required a structural growth. On dataset A (4 input dimensions), this structural growth was not sufficient to maintain 100% accuracy in performance. The best one-layer network was composed of 12 units in the internal layer and performed with maximum accuracy of 77.18%, whereas the best hidden-layer network, in terms of maximum accuracy, was composed of 19 units in internal layer and 11 units in hidden layer and performed with the maximum accuracy of 85.57%. Table 2 summarizes the results concerning the best three selected one-layer and hidden-layer networks for solving formal-stage-11 problems on dataset A. These networks were selected based on the value of accuracy and on a value of differential efficiency. Only values within the same magnitude range were selected.

**Table 2:** Networks solving formal-stage-11 problems with dataset A.

Network type	Diff EF	Units (internal layer)	Units (hidden layer)	Nr. Connections	Mean Accuracy (%) (∓ sd)	Max accuracy (%)
**One-Layer networks**	-5.95	12	—	99	73.60 (± 2.43)	77.18
	-1,47	3	—	27	59.85 (± 7.86)	67.15
	-0,04	20	—	163	74.93 (± 2.79)	79.37
**Hidden-layer network**	-418,12	14	7	199	67.85 (∓4,16)	81.32
	-208,51	10	9	179	67.13 (∓3.67)	77.41
	-198,36	19	11	351	68.90 (∓10.70)	85.57

Table contains Structure, Mean Accuracy, Standard Deviation, and Maximum Accuracy of selected Networks for solving Formal-stage-11 problems with dataset A

On dataset B (8 input dimensions), the structural growth allowed to achieve 100% accuracy with a one-layer network composed of 12 units. One reason for this is that the increased number of dimensions of dataset B allows for encoding the input in a more distributed manner, demonstrating the distributive potential of neural networks. However, the best hidden-layer network was composed of 17 units on the internal layer and 2 units on the hidden layer, and performed with a maximum accuracy of 99.20%, with a mean accuracy of 50.73% (∓25.42). This highlighting the instability of learning in a network with an increased number of degrees of freedom and the balance that is required to achieve between the distributive potential of neural networks and the information contained in the input set.

In regards to systematic-stage-12 problems, only tested with dataset B, network performance slightly dropped to 90.91%, with 11 units in internal layer and 8 units in hidden layer, or with 18 units in the internal layer and 10 in the hidden layer. Table 3 summarises the results concerning the selected one-layer and hidden-layer networks for solving Systematic-stage-12 problems, based on the accuracy and on the value of differential efficiency within the same magnitude range.

**Table 3:** Networks solving solving systematic-stage-12 problems with dataset B.

Network type	Diff EF	Units (internal layer)	Units (hidden layer)	Nr. Connections	Mean Accuracy (%)	Max Accuracy (%)
**One-layer network**	-18.72	20	—	243	63,82 (∓8,14)	75,68
	-14.89	16	—	195	80,70 (∓3,45)	86,17
**Hidden-layer network**	-934.9	11	8	222	61,84 (∓27,51)	90,91
	-567	17	13	429	67,72 (∓26,16)	92,93
	-474.8	18	10	385	62,58 (∓26,58)	90,91

Table contains Structure, Mean Accuracy, Standard Deviation, and Maximum Accuracy of selected Networks for solving Systematic-stage-12 problems with dataset B

The highlights of these results concern the following topics: for the feedforward connectivity pattern, concrete-stage-9 and abstract-stage-10 problems were easily solved by a perceptron network with 100% accuracy, which was expected, due to the fact that these problems required count and sum operations and a neural network operates based on a weighted sum of inputs throughout its structure. In regards to formal-stage-11, in order to maintain a 100% accuracy, it was necessary to use dataset B and to grow the network structure in the internal layer up to 12 units. When transiting for systematic problems, it was necessary that the network grew another layer of units to maintain performance accuracy near the maximum, even though a 100% could not be achieved in any trial. Furthermore, the maximum value of 99.20% accuracy was an outcome of a rare combination of connection weights, highlighting the huge performance variability and instability of hidden layer networks for solving these formal problems. Actually, results show that the more complex the problem is and the more complex the structure is, the more variable the performance, with the standard deviation values increasing by one order of magnitude from the formal to the systematic problem solving, and from perceptron to hidden layer networks, respectively.

**Fig 15:**
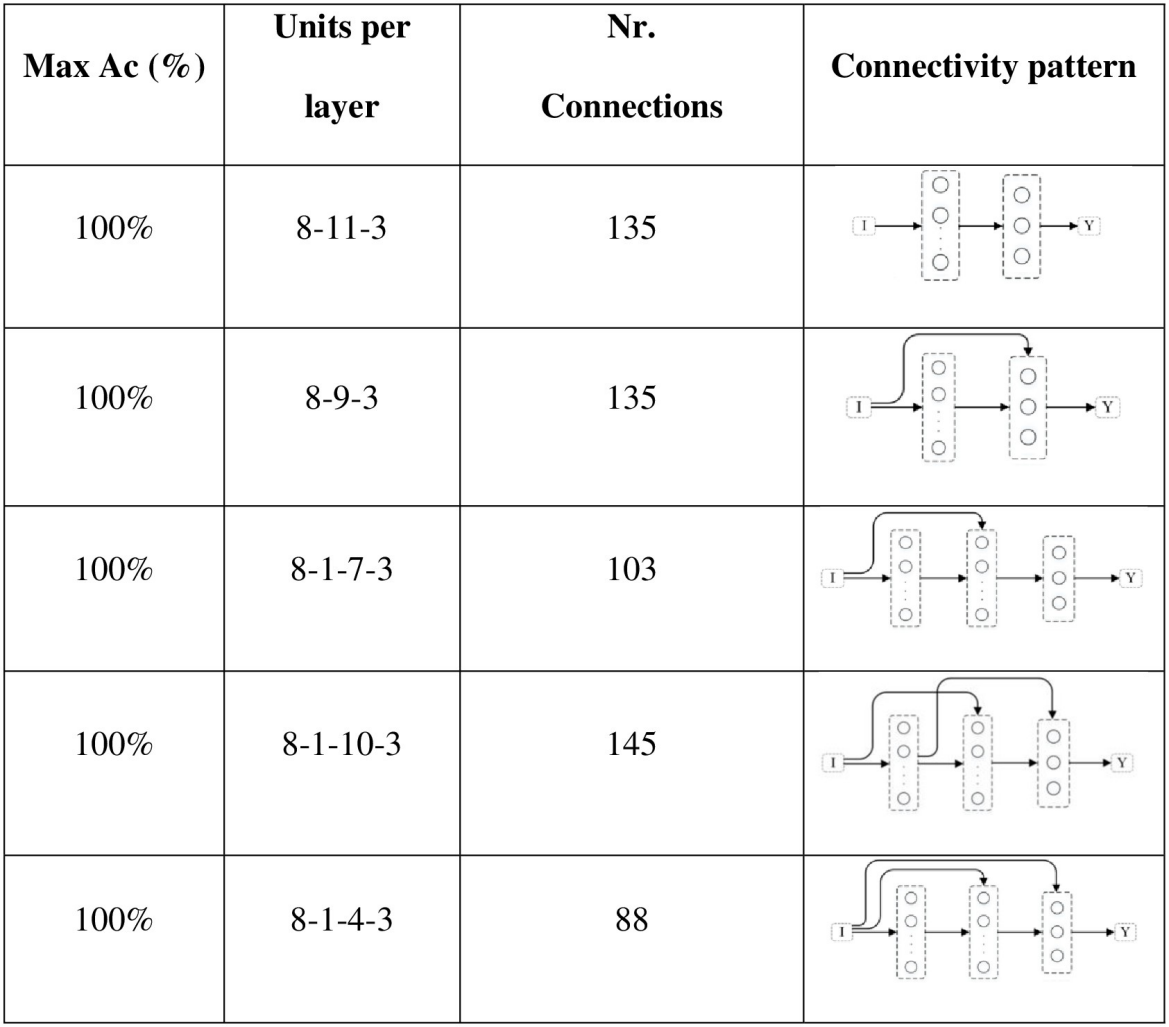
Network structure and connectivity pattern for formal-stage-11 problem solving. The second column of the table should be read as following: 8-11-3 means that the network had 8 units in the input layer, 11 units in the internal layer and 3 units in the output layer; 8-1-10-3 means that the network had 8 input entries, 1 unit in the internal layer, 10 units in the hidden layer, and again 3 output units.

When testing different connectivity patterns, only formal-stage-11 and systematic-stage-12 sub-problems were trained with dataset B. Fig 15 shows that, with different connectivity patterns, one layer and hidden layer networks solved formal-stage-11 problems with 100% accuracy. All these successful connectivity patterns shared the common characteristic of input units being linked to units in subsequent layers. We term this connectivity pattern as input connectivity. Interestingly, network 7, the network with densest input connectivity (Fig 11) required the least number of connections, which might explain why manipulating the connectivity pattern allowed to improve performance accuracy and decrease performance instability.

**Fig 16:**
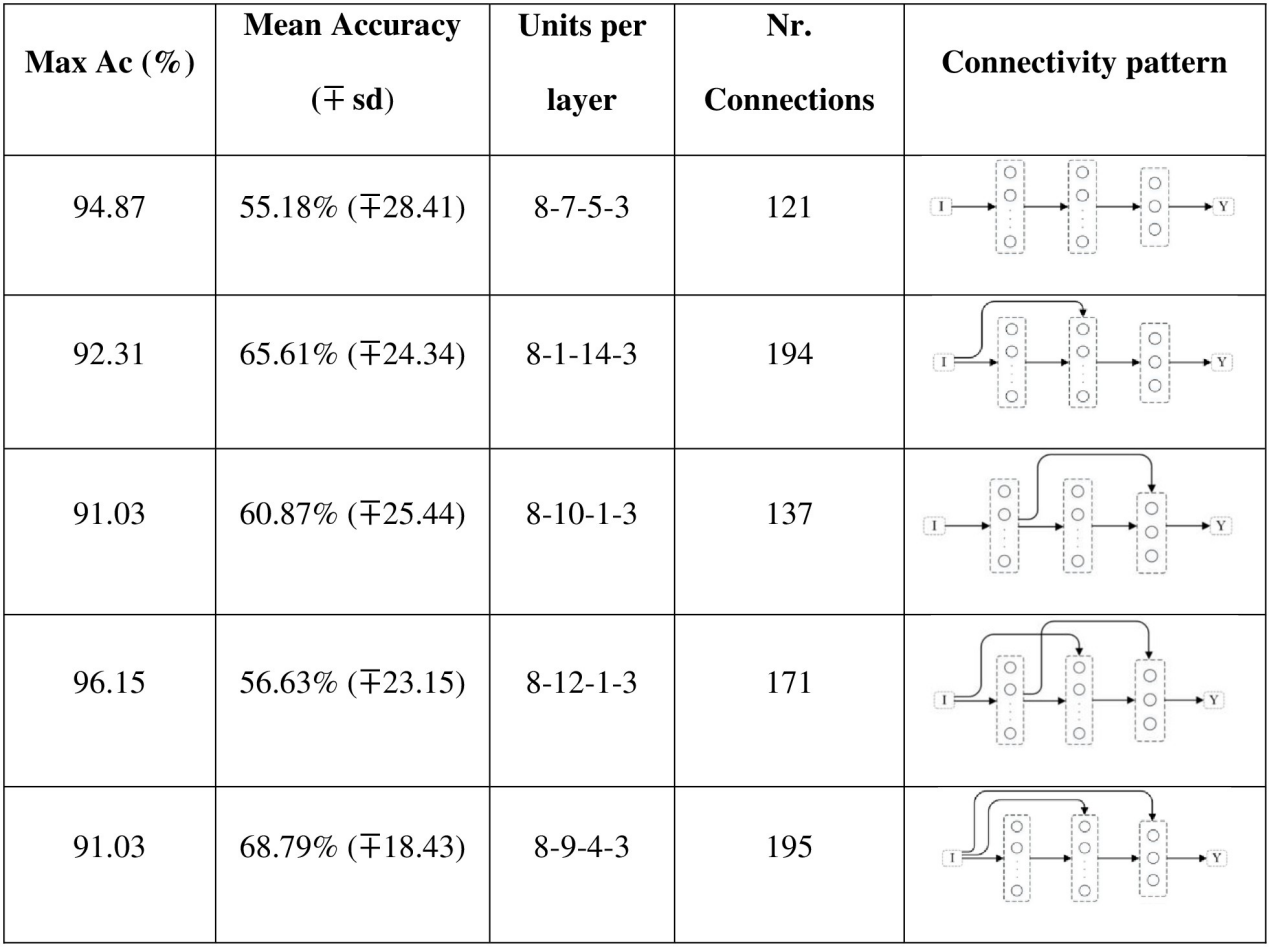
Network structure and connectivity pattern for systematic-stage-12 problem solving. The second column should be read following the same rules as Fig 15.

In regards to systematic-stage-12 sub-problems, Fig 16 shows that the network structure always required to be a hidden layer network, but, again, like in the case of a feedforward connectivity, none was able to solve the set of sub-problems with 100% accuracy and all kept a standard deviation of two orders of magnitude. It is also important to note that two of the five networks converged to approximately the same number of connection (194 and 195).

**Fig 17:**
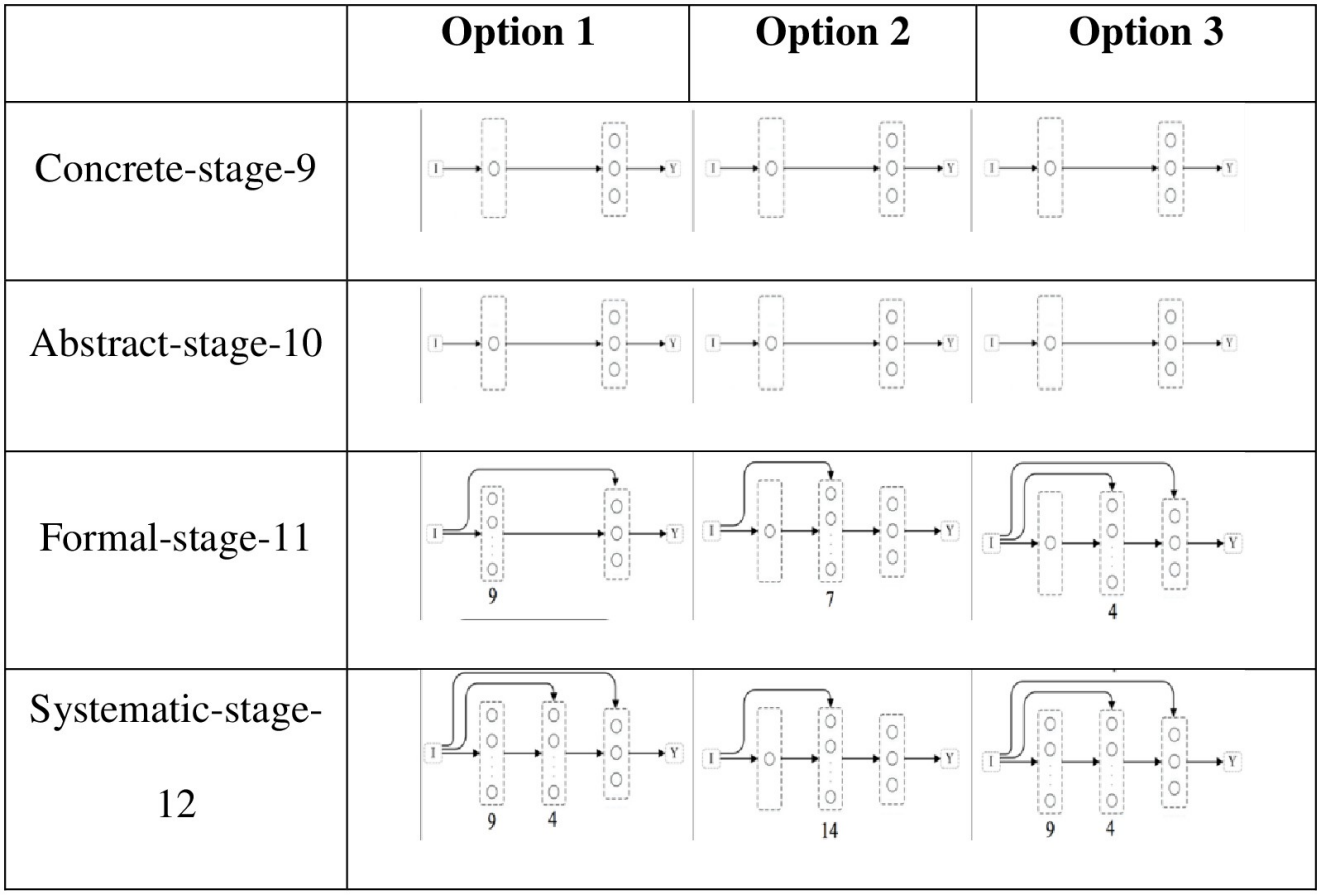
Possible network progressions. Table representing transitions from concrete to systematic problem-solving. Each column represents a possible network structure growth throughout the four tested OHC, represented in lines.

Given the above selected best performing networks for each OHC sub-problem, the next step was to re-select some, based on how their components (layers, units and connections) could be hierarchically organized. This hierarchical organization pretends to show how networks can grow structurally to accommodate increasingly complex problem solving. Fig 17 shows three possibilities of structural growth. Given the results stated above, the structure of networks across options (columns) does not vary in stages 9 and 10 (rows 1 and 2). In option 1, from abstract to formal, the network grows 8 units in the internal layer and, then, from formal to systematic, it grows 4 more units in the hidden layer; in option 2, the network grows a hidden layer from abstract to formal and, from formal to systematic, it doubles the amount of units I the hidden layer; in option 3, the network first grows a hidden layer from abstract to formal and, from formal to systematic, it maintains the hidden layer and grows the double of the number of units in the internal layer, which it has grew before in the hidden layer.

Finally, given the hierarchical organization of the best performing networks, we tested whether lower-order networks could be protected from the interference of higher-order networks. This was done by testing different conditions of learning rate (LR) over the final weight matrices of the immediately lower-order network. In the transition from concrete-stage-9 to abstract-stage-10 sub-problems, results show that independently of the LR condition, the networks always learned with 100% accuracy. In the remaining transitions, from abstract to formal (10–11) and from formal to systematic (11–12), performance slightly dropped. Table 4 shows the performance of each network after the transition, for the three options selected above in the remaining OHC.

**Table 4:** Sequential network performance across orders.

LR	OHC	Option 1	Option 2	Option 3
**100%**	**10–11**	98.72%	94.87%	96.15%
	**11–12**	97.44%	87.18%	96.15%
**<100%**	**10–11**	97.44%	87.18%	96.15%
	**11–12**	97.44%	87.18%	96.15%
**0**	**10–11**	97.44%	93.59%	98.72%
	**11–12**	85.90%	91.03%	85.90%

Network performance across progression with different learning rates

Fixing the weights of abstract-stage-10 networks slightly negatively influenced formal-stage-11 networks performance, since that none achieved a 100% accuracy as when they were initialized with random weights. LR modifications did not impact the results in a significant manner, although they show that there is a tendency to decrease in accuracy as the LR is decreased, as expected. Each network has only been trained on the datasets of the correspondent complexity that created them.

## Discussion

The first objective of this work was to explore whether a neural network model would exhibit a complexity structural growth to solve tasks of increasing hierarchical complexity. For this, the input set was segregated into subtasks of increasing complexity, measured according to their Order of Hierarchical Complexity (OHC). There is evidence that the OHC is a reliable indicator of task complexity and a reliable indicator of the underlying problem-solving structure necessary to solve the tasks [11, 19].

Results confirm that the neural network models captured the increase in hierarchical complexity of inputs, shown by the increase in their structural complexity. Each hierarchical transition in problem solving required a structural adaptation of neural networks. These adaptations varied in terms of number of units, number of connections, and connectivity pattern among layers. At the end, three network structural progressions could be identified across orders of hierarchical complexity problems. The fact that three different structural progressions were found that performed with similar accuracy is aligned with the idea of self-organization [5, 7, 10, 11, 65, 66], where a system accommodates more elements to respond to a more complex situation by reorganizing its internal structure as a whole [67]. In terms of cognitive performance and brain organization, the idea of self-organization is also associated with the idea of inter-variability [15, 16], suggested by the results of this study considering the three ways of internal organization and optimization for achieving the same outputs.

Concerning these three options and how they evolve across orders of complexity, there is a parallelism in terms of the magnitude of the models. For concrete and abstract problems, models contained one unit in the internal layer and slightly varying number of active connections. For formal problems, models recruited more than one unit in the internal layer and benefitted from a denser connectivity pattern among layers. Specifically, a denser connectivity pattern improved performance of hidden networks solving formal problems, even though the connectivity pattern of models for solving formal problems varied across the three selected options. Within this variation, one should note that the densest connectivity pattern (option 3) led to the recruitment of fewer elements, both units and connections. This aligns with the fact that the intricate connectivity pattern of the brain allows it to produce immensely more complex outputs than a computer simulation, although it has fewer and slower connections than are usually used in a computer simulation. For systematic problems, models always recruited two layers of units, even though at the expense of an accuracy decrease. Also, it is interesting to note that the two different options of models for solving systematic problems converged to the practically the same number of connections (194 and 195) although with different number of units, pointing out that dependencies among subparts can be seen as elements of the system. Since the OHC can be applied to all tasks and to all agents that solve tasks, these results demonstrate that the OHC is a promising measure to apply to input segregation in different datasets so as to explore the behavior of systems across different datasets. Indeed, it is very useful to find a common research ground to explore the potential of neural networks to be simulators of the structural growth observed throughout development and evolution.

The second objective was to test whether it was possible to generate a higher-order structure departing from the weights of the lower-order structure without overwriting the lower-order structure. This relates to the problem of catastrophic forgetting, which refers to the fact that when a neural network learns a new problem, or a more complex version of the same problem, previous weights get overwritten by the requirements of the new task. Hence, previous tasks are forgotten to a point where the network can no longer solve them [55]. When we talk about hierarchical catastrophic forgetting, we are referring to the fact that when a higher order task is learnt, the weights for a lower-order task get overwritten and the model begins to employ the more complex version to solve simpler and more complex versions of the task, as occurs in generative algorithms [52]. For this reason, we did not test each higher order structure on lower order input sets. Previous work in the literature experimented with different forms of presenting developmental inputs to the network, such as presenting all inputs together in a batch, showing interesting forms of network arrangement, but all conducting to the problem of hierarchical catastrophic forgetting or to a network performance which was poor on the multiplicative (formal) subsets of the balance beam problem [46, 47, 68]. By segregating inputs by OHC and looking for a network structure responsible for each OHC, this work was successful in using input segregation as an architectural method for mitigating catastrophic forgetting [59], even though there was a small decrease in accuracy of the higher-order model when sequential models were trained sequentially.

When comparing the results of these simulations with previous network simulations in the balance beam problem, some advantages of this segregation method have been found. First, for the first time, multiplicative problems, also called Torque-Difference problems, have been solved with an accuracy of 100%, differently from previous experiments [46, 68, 69]. This clearly shows the relevance of segregating problem-solving structures by the order of complexity of inputs. Second, no previous experiment has tested the latest subset of complexity balance scale problems, systematic-stage-12 problems. Third, this was the first time that lower-order structures could remain available after higher-order structures were created, which is a crucial aspect of development and concretizes a successful approach to the problem of catastrophic forgetting [5, 55]. In fact, addressing developmental problem solving in neural networks implies addressing hierarchical catastrophic forgetting. It is an important aspect of development that a person can always go down to more elementary levels of problem solving [5].

Finally, even though the tasks presented by the balance beam problem are apparently very simple tasks to be performed by a machine learning model, the fact, so far, is that it has been difficult to reproduce human performance on this test with an algorithm [37, 48, 68, 69]. First, algorithms are data-driven, based on associations between dimensions, whereas the balance beam problem requires that operations among dimensions are conducted [34]. Second, in order to track the developmental progress of the structure of a neural network model during task performance, and given that neural networks are considered the classic black boxes of machine learning, this work opted for working with a dataset that could easily be tracked and solved by a human, and which could offer insights into how to build a developmental neural network model.

This work also contains some limitations that we intend to address in future work. First, pruning methods have not been included in the search for the minimal network model. Second, since the number of hyper-parameters that influence learning in a network model is huge, finding the optimal neural network structure for solving problems can be difficult to replicate. Third, the operations of the concrete and abstract OHC problems conflicts with the mathematical formalization of units in a neural network, which drastically influenced the structure of the correspondent models. Nonetheless, a progression was found in further orders of complexity, not compromising the overall picture of results.

## Conclusion

Hierarchy is evidenced in the brain throughout development and evolution. This work aimed at observing the existence of a hierarchical organization in the structure of a neural network model that was trained on increasingly complex problems. In order to do that, for the first time in the literature, the input set was segregated by orders of hierarchical complexity (OHC), as defined by the Model of Hierarchical Complexity (MHC). Even with networks being trained separately on each increasingly complex input subset, three structural progressions were found across complexity samples. This shows that neural network models grasped the complexity structure present in the MHC, and pointed towards the principles of self-organization and inter-variability. Also, in all these progressions, a higher order structure did not replace the lower order structure, which includes this methodology in the class of architectural-based methodologies for successfully addressing the problem of catastrophic forgetting. Segregating inputs by OHC has the main advantage of allowing for a systematic formalization of knowledge in terms of using neural networks as simulators of structural hierarchical growth, since all tasks presented in all datasets can be attributed an OHC. In future work, within the context of the balance beam test, addressing both simpler and more complex configurations below the concrete stage and beyond the systematic stage is envisioned. An example of an order of hierarchical complexity above the systematic stage (meta-systematic stage 13) is one that requires the use of quadratic sum. It would also be interesting to test the same input segregation methodology with different algorithms to observe whether the increase in complexity is, and how it is, universal across machine learning models. In this case, a measure of computational complexity, such as the Kolmogorov complexity, could be used to address complexity transversally across models. Also, aligned with the advantages of using the rationale of the Model of Hierarchical Complexity, the same input methodology is intended to be applied to different datasets to replicate and deeper understand the structural growth observed in neural network models. Different datasets will certainly concern more complex tasks to solve in terms of operations and correlations among dimensions. Finally, future work is intended to address and quantify the question of catastrophic forgetting and complexity transition in more detail, such as the automatization of criteria for network selection and the mechanisms for triggering network structural growth as more complex problems are presented to it.

## Supporting information

S1 Dataset (ZIP)
