# Correction: Hierarchical growth in neural networks structure: Organizing inputs by Order of Hierarchical Complexity

**DOI:** 10.1371/journal.pone.0308115

**Published:** 2024-07-25

**Authors:** Sofia Leite, Bruno Mota, António Ramos Silva, Michael Lamport Commons, Patrice Marie Miller, Pedro Pereira Rodrigues

There are errors in the author affiliations. The correct affiliations are as follows:

Sofia Leite^1,2^, Bruno Mota^3^, António Ramos Silva^4,5^, Michael Lamport Commons^2,6^, Patrice Marie Miller^2,7^, Pedro Pereira Rodrigues^1,8^

1 CINTESIS@RISE—Center of Health Technologies and Services Research at Health Research Network, **2** Dare Association, Inc. Boston, Massachusetts, United States of America, **3** Laboratory of Experimental Mathematics and Theoretical Biology, Physics Institute, Universidade Federal do Rio de Janeiro, Rio de Janeiro, Brasil, **4** Department of Mechanical Engineering, Faculty of Engineering University of Porto, Porto, Portugal, **5** INEGI Institute of Science and Innovation in Mechanical and Industrial Engineering, Porto, Portugal, **6** Beth Israel Deaconess Medical Center, Harvard Medical School, Cambridge, Massachusetts, United States of America, **7** Department of Psychology, Salem State University, Salem, Massachusetts, United States of America, **8** Faculty of Medicine, University of Porto
